# Chewing with a cleft palate: a randomized controlled trial. Effect of timing of surgical closure of the palate on mastication in infants with clefts

**DOI:** 10.1007/s00784-025-06467-2

**Published:** 2025-09-02

**Authors:** C. H. A. L. Guillaume, M. M. R. Verhoeven, A. M. Eligh, S. J. Haverkamp, R. M. J. C. Eijkemans, M. Kon, A. B. Mink van der Molen, C. C. Breugem

**Affiliations:** 1 Department of Pediatric Plastic Surgery, Wilhelmina Children’s Hospital, University Medical Center Utrecht, Utrecht, The Netherlands; 2 Department of Pediatric Plastic Surgery, Emma Children’s Hospital, Amsterdam University Medical Center, Amsterdam, The Netherlands; 3 Department of Speech Therapy, Wilhelmina Children’s Hospital, University Medical Center Utrecht, Utrecht, The Netherlands; 4 Department of Biostatistics and Research Support, Julius Centre, University Medical Centre, Utrecht, the Netherlands

**Keywords:** Oral clefts, Cleft lip, Cleft palate, Mastication, Mastication observation and evaluation score, MOE

## Abstract

**Objectives:**

To evaluate the effect of surgical timing of palatal closure on overall masticatory function in infants with cleft lip and/or palate (CL/P).

**Materials and methods:**

In this randomized controlled trial, 60 children with cleft lip and/or palate (CL/P) were assessed at the Wilhelmina Children’s Hospital, University Medical Center Utrecht, the Netherlands. Participants were randomly assigned to early closure (Group A: 6–8 months) or late closure (Group B: 10–12 months). Chewing function was evaluated at 9, 13, and 17 months of age using the Mastication Observation and Evaluation (MOE) instrument, which scores eight components of mastication.

**Results:**

No statistically significant differences were found in total MOE scores between groups at any time point. However, at 17 months, Group B showed significantly better lateral tongue movement than Group A (mean = 2.72, SD = 0.53 vs. mean = 2.45, SD = 0.51; *p* = .047).

**Conclusions:**

Early palatal closure (6–8 months) does not improve overall mastication compared to late closure (10–12 months) in the first 17 months of life.

**Clinical Relevance:**

Research on mastication in children with CL/P is limited. This study provides novel insights into functional outcomes of palatal surgery, supporting evidence-based refinement of cleft treatment protocols.

**Trial Registration Number:**

The CLEFED study was approved by the Medical Ethics Committee of the University Medical Center Utrecht, The Netherlands (registration no: NTR3275). Date of registration: November 2011. Trial Register: https://www.trialregister.nl/trial/3125 (trial number: NTR3275)

## Introduction

Oral clefts are among the most common craniofacial malformations. In the Netherlands, the prevalence of cleft lip and/or palate (CL/P) between 1977 and 2007 was 16.6 per 10,000 live births [1, 2], corresponding to approximately 315–350 affected infants born each year [1]. Globally, incidence rates vary considerably depending on factors such as ethnicity, geographic location, and socioeconomic factors [3].

Clefts may involve the lip, the palate, or both, and can differ in location and severity. Palatal clefts are associated with feeding difficulties, speech impairments, and altered dentofacial development [4]. Up to 67% of infants with cleft palates experience feeding problems, though estimates vary across populations and clinical contexts [5].

Mastication, or chewing, is a crucial part of feeding. It is a complex process that transforms food into a swallowable bolus. During mastication, the tongue, lips, and cheeks guide food to the molars, where it is broken down by the teeth and jaws [6, 7]. As food is processed, the resulting bolus is moved posteriorly for swallowing [6]. Mastication typically begins around 6 months of age, when infants transition from sucking to chewing. By 12 months, most typically developing children can manage a variety of food textures [6]. Proper mastication reduces the risk of choking by ensuring that food is adequately fragmented. It also stimulates saliva production, which plays a protective role in oral health. Coordination and strength in mastication continue to improve until about 4 years of age, enabling children to handle increasingly complex foods [6]. This development is shaped by both behavioral factors and the biomechanics of chewing and swallowing [7].

In children with clefts, altered orofacial anatomy can impair mastication. Prior studies have explored various aspects of mastication in this population, including anterior crossbite [8], unilateral chewing patterns [8], masticatory muscle activity [9], and craniofacial growth patterns [10]. Evidence suggests that children with CL/P often demonstrate altered masticatory muscle activation and reduced mandibular rotation, leading to inefficient food breakdown [9]. Over time, these functional impairments may contribute to long-term skeletal adaptations [11].

At the Wilhelmina Children’s Hospital (WCH) in Utrecht, the Netherlands, palatal closure is typically performed between 10 and 12 months of age. However, the optimal timing of this procedure remains a topic of debate, and no universally accepted guidelines exist. Previous studies have examined the effect of surgical timing on speech outcomes [12–17], hearing [13, 14], maxillary growth [12–14, 18–24] and complications [13, 14, 16, 17, 25].

Despite this body of research, limited information is available regarding the influence of surgical timing on the development of mastication. This randomized controlled trial aims to investigate whether earlier palatal closure (at 6–8 months) results in improved masticatory function compared to later closure (at 10–12 months). We hypothesize that early surgical intervention is associated with better masticatory development in children with cleft palate.

## Methods

### Participants

This study is part of the CLEfts and FEeding Difficulties (CLEFED-1) research project, a randomized controlled trial initiated in 2012 to investigate the impact of surgical timing on feeding outcomes in children with cleft palate. Participants were consecutively recruited between 2012 and 2017 from the outpatient clinic of the cleft team at Wilhelmina Children’s Hospital (WCH), Utrecht, the Netherlands.

Inclusion criteria were: (1) cleft palate only or cleft lip and palate, (2) informed parental consent, and (3) sufficient parental proficiency in Dutch. Exclusion criteria included adoption, the presence of syndromes or additional diagnoses affecting orofacial function, and insufficient parental understanding of Dutch language.

The study was approved by the Medical Ethics Committee of the University Medical Center Utrecht (registration no. NTR3275). Participants were randomly assigned to one of two groups using a computer-generated allocation schedule:

- Group A: palatal closure at 6–8 months.
- Group B: palatal closure at 10–12 months.

Of the 80 children originally enrolled, 60 were included in the mastication assessments: 30 in Group A and 30 in Group B. The reduced number of assessments was due to the discontinuation of funding for home visits toward the end of the study, which made it logistically unfeasible to complete all planned assessments.

Due to the nature of the intervention, blinding of surgeons and caregivers was not feasible. However, video-based mastication assessments were independently scored by speech-language therapists who were blinded to group allocation. The principles of informed consent, autonomy, and equal access to care were fully upheld; parents were assured that declining participation would not affect the care their child received.

### Measures

The Mastication Observation and Evaluation (MOE) instrument, developed by *Remijn et al.* [6], was used to structure and standardize the clinical observation of chewing in children. The MOE assesses eight aspects of chewing: tongue protrusion, lateral tongue movement, munching, jaw movement, chewing duration, loss of food or saliva, number of swallows, fluency and coordination. Each item is scored on a 4-point ordinal scale ranging from ‘very inappropriate’ to ‘very appropriate,’ yielding a maximum total score of 32. Scores of 1 or 2 indicate insufficient mastication, while scores of 3 or 4 are considered sufficient. The MOE has been validated for use in typically developing children aged 0.5–4 years and in children with cerebral palsy aged 2–6 years [6].

### Procedure

Mastication was assessed at three fixed ages: 9 months (M1), 13 months (M2), and 17 months (M3). This approach allowed the study to capture developmental changes independent of postoperative timing. Group A had already undergone palatal closure by M1. All assessments were conducted during home visits by two trained speech-language therapists from the WCH cleft team.

At each assessment, children were offered three equal-sized pieces of solid food (e.g., bread cubes of approximately 1 cm³). A new piece was presented only after the previous one had been fully consumed. Sessions were recorded using a digital camcorder with a hard-disk drive. To be included in the analysis, each video needed to show at least three clearly visible bites. Food types such as bananas, crackers, or cookies were excluded to ensure consistency. Videos were scored independently by both speech-language therapists. Discrepancies were resolved through discussion and consensus.

### Surgical procedure

The surgical procedures were performed by two highly experienced cleft surgeons using a standardized technique. At approximately 3 months of age, all participants with CL/P underwent lip closure combined with a vomer flap.

For children with soft palate clefts only, the palate was closed using an intravelar veloplasty with a straight-line repair. In cases involving both the soft and hard palate, closure was achieved using intravelar veloplasty in combination with the von Langenbeck technique.

### Statistical analysis

Statistical analyses were conducted using SPSS version 26. Descriptive statistics (medians and ranges) were used to summarize patient characteristics and MOE scores. Normality of data was assessed using the Kolmogorov–Smirnov test and inspection of boxplots.

To compare total MOE scores between Groups A and B at each time point (M1, M2, M3), independent samples t-tests were performed. Individual MOE items were analyzed separately using the same test to explore potential functional differences. A significance level of α = 0.05 was applied to all analyses.

## Results

### Patient characteristics

Mastication assessments were conducted in 60 children, of whom 29 were girls. Group A (early closure) and Group B (late closure) each included 30 children (14 and 15 girls, respectively). Participant characteristics for this subsample are summarized in Table 1. No significant differences were observed in gender distribution (*p* =.796), cleft classification (*p* =.125), cleft severity (*p* =.425), or birthweight (*p* =.375). However, the incidence of fistula surgery differed significantly between the groups (*p* =.040), with more children in Group B requiring surgical repair.

**Table 1:** Demographic data and participant characteristics of study population

	Group A (palatal closure age 6–8 months)	Group B (palatal closure age 10–12 months)	
Total	*n* = 30	*n* = 30	
Gender			*p* =.796
Male	*16 (53.3%)*	*15 (50%)*	
Female	*14 (46.7%)*	*15 (50%)*	
Cleft type			*p =.125*
CLP right	*5 (16.7%)*	*2 (6.7%)*	
CLP left	*14 (46.7%)*	*8 (26.7%)*	
CLP bilateral	*4 (13.3%)*	*9 (30%)*	
CP	*7 (23.3%)*	*11 (36.7%)*	
Extent of the cleft			*p* =.425
Type 0	*2 (6.7%)*	*0 (0%)*	
Type 1	*1 (3.3%)*	*3 (10%)*	
Type 2	*10 (33.3%)*	*9 (30%)*	
Type 3	*7 (23.3%)*	*5 (16.7%)*	
Type 4	*8 (26.7%)*	*11 (36.7%)*	
Missing	*2 (6.7%)*	*2 (6.7%)*	
Mean age in weeks (SD)			
M1*	*39.35 (1.37)*	*39.34 (1.11)*	
M2*	*56.36 (1.47)*	*57.15 (1.21)*	
M3*	*74.28 (1.13)*	*74.63 (1.19)*	
Mean length in cm (SD)			
M1	*71.78 (2.52)*	*71.99 (3.04)*	
M2	*76.81 (2.68)*	*74.53 (13.21)*	
M3	*81.22 (2.76)*	*81.67 (3.13)*	
Mean birthweight in kg (SD)	*3.488 (0.424)*	*3.353 (0.707)*	*p* =.375
Mean weight in kg (SD)			
M1	*8.495 (0.941)*	*8.352 (0.996)*	
M2	*9.861 (1.013)*	*9.509 (1.031)*	
M3	*11.016 (1.012)*	*10.739 (1.217)*	

*M1 = measurement 1 (9 months), M2 = measurement 2 (13 months), M3 = measurement 3 (17 months)

### Mastication age 9 months

At 9 months, valid MOE scores were available for 22 children in Group A. The remaining eight recordings were excluded due to missing videos (*n* = 3), poor video quality (*n* = 1), inappropriate food consistency (*n* = 3), or refusal to eat (*n* = 1). In Group B, 29 valid recordings were available; one recording was missing.

**Fig. 1:**
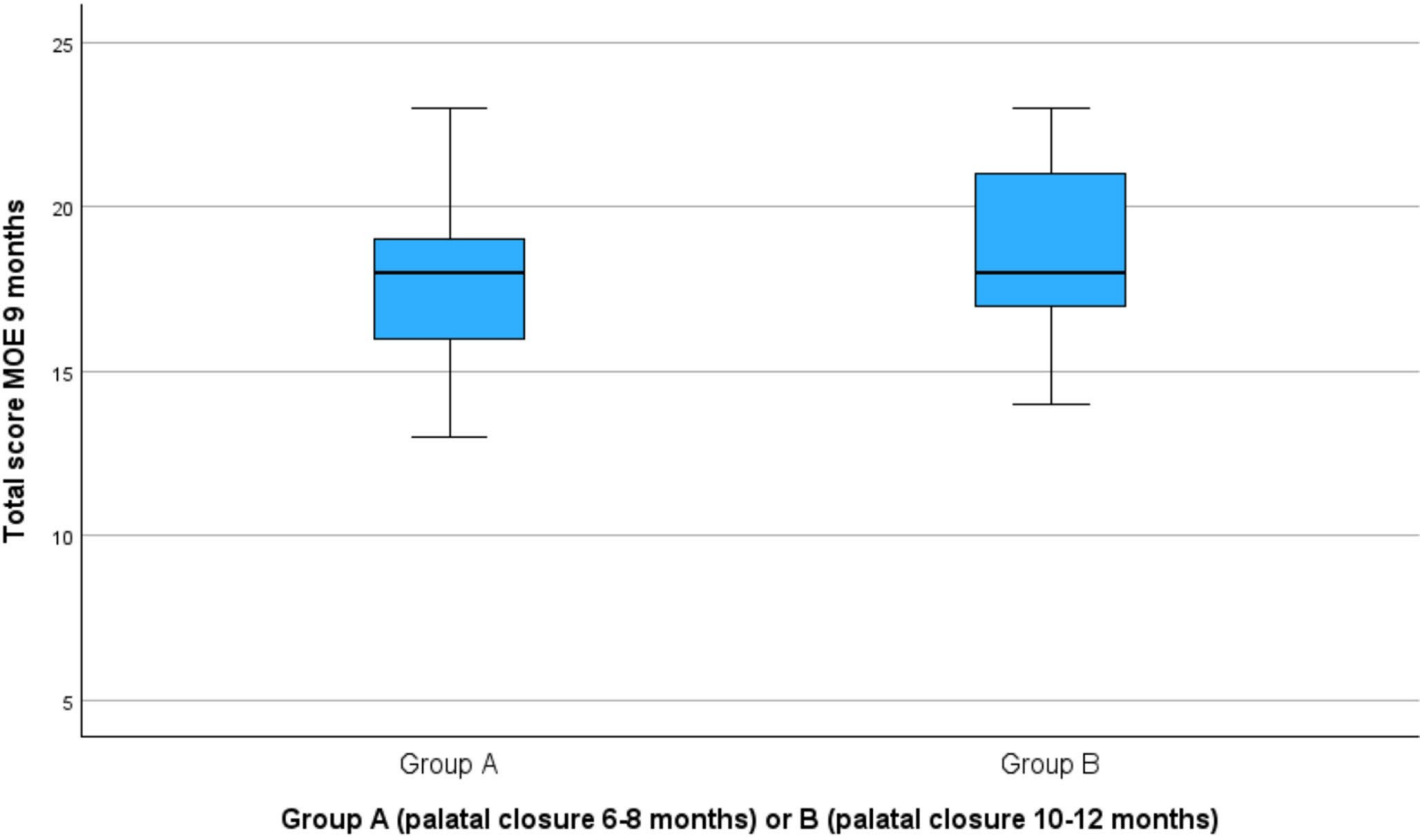
Distribution total MOE score of group A (*n* = 22) and B (*n* = 29) at the age of 9 months

Total MOE scores ranged from 8 to 23 in both groups (Fig. 1). The mean total MOE score was 17.77 (SD = 3.19) in Group A and 18.24 (SD = 3.52) in Group B. An independent samples t-test revealed no significant difference between the groups (t(47) = −0.50, *p* =.622), with a mean difference of −0.47 and a 95% confidence interval (CI) of [−2.37, 1.43]. No significant differences were found in any individual MOE items.

### Mastication age 13 months

At 13 months, valid MOE scores were obtained for 26 children in Group A and 27 in Group B. In Group A, three recordings were missing, and one child did not chew the food. In Group B, three recordings were missing.

**Fig. 2:**
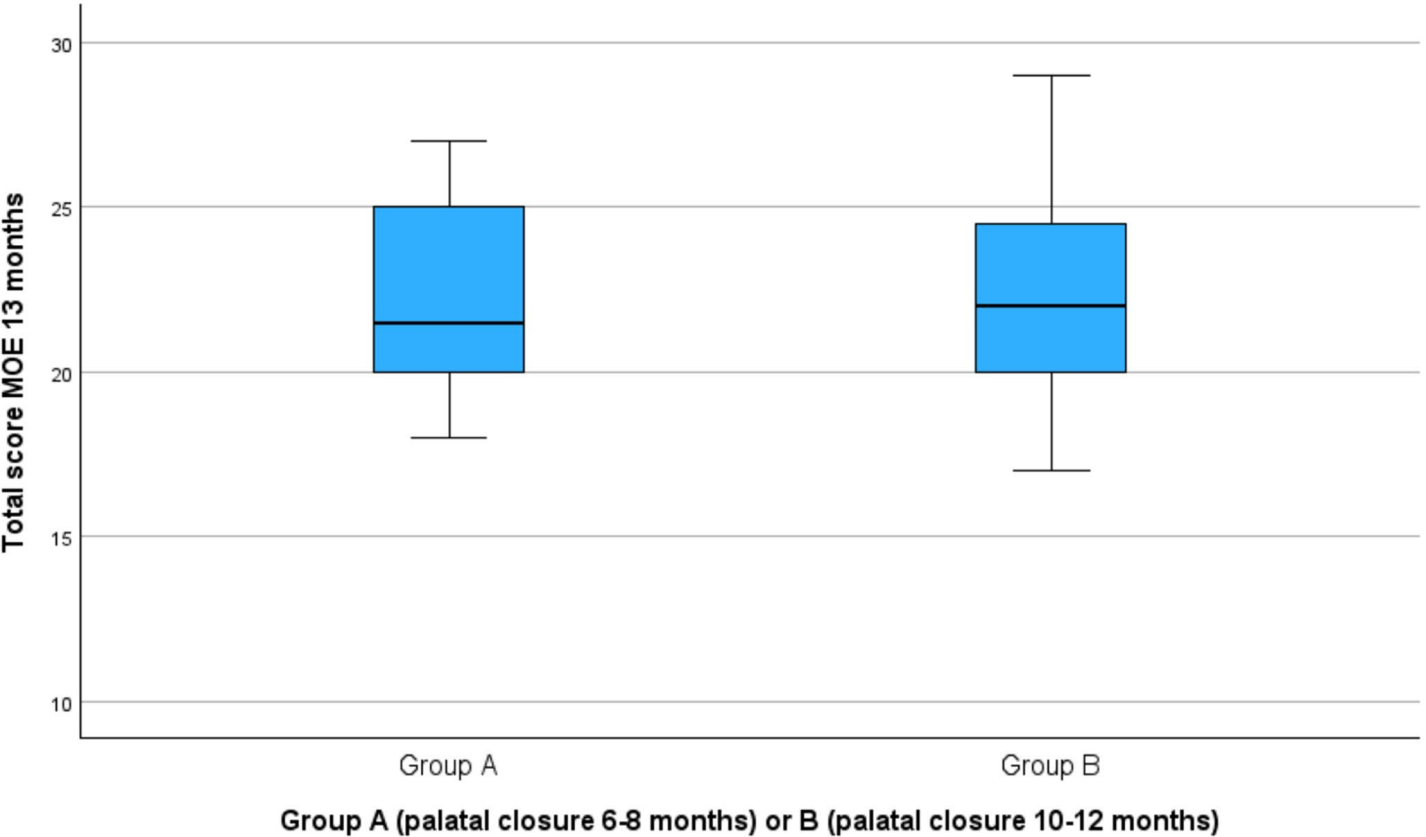
Distribution total MOE score of group A (*n* = 26) and B (*n* = 27) at the age of 13 months

Total MOE scores ranged from 18 to 27 in Group A and from 13 to 29 in Group B (Fig. 2). The mean total MOE scores were 22.12 (SD = 2.88) in Group A and 22.11 (SD = 3.68) in Group B. No significant difference was found between groups (t(49) = 0.005, *p* =.996), with a mean difference of 0.004 (95% CI [−1.82, 1.82]). Individual MOE item scores did not differ significantly between groups.

### Mastication age 17 months

**Fig. 3:**
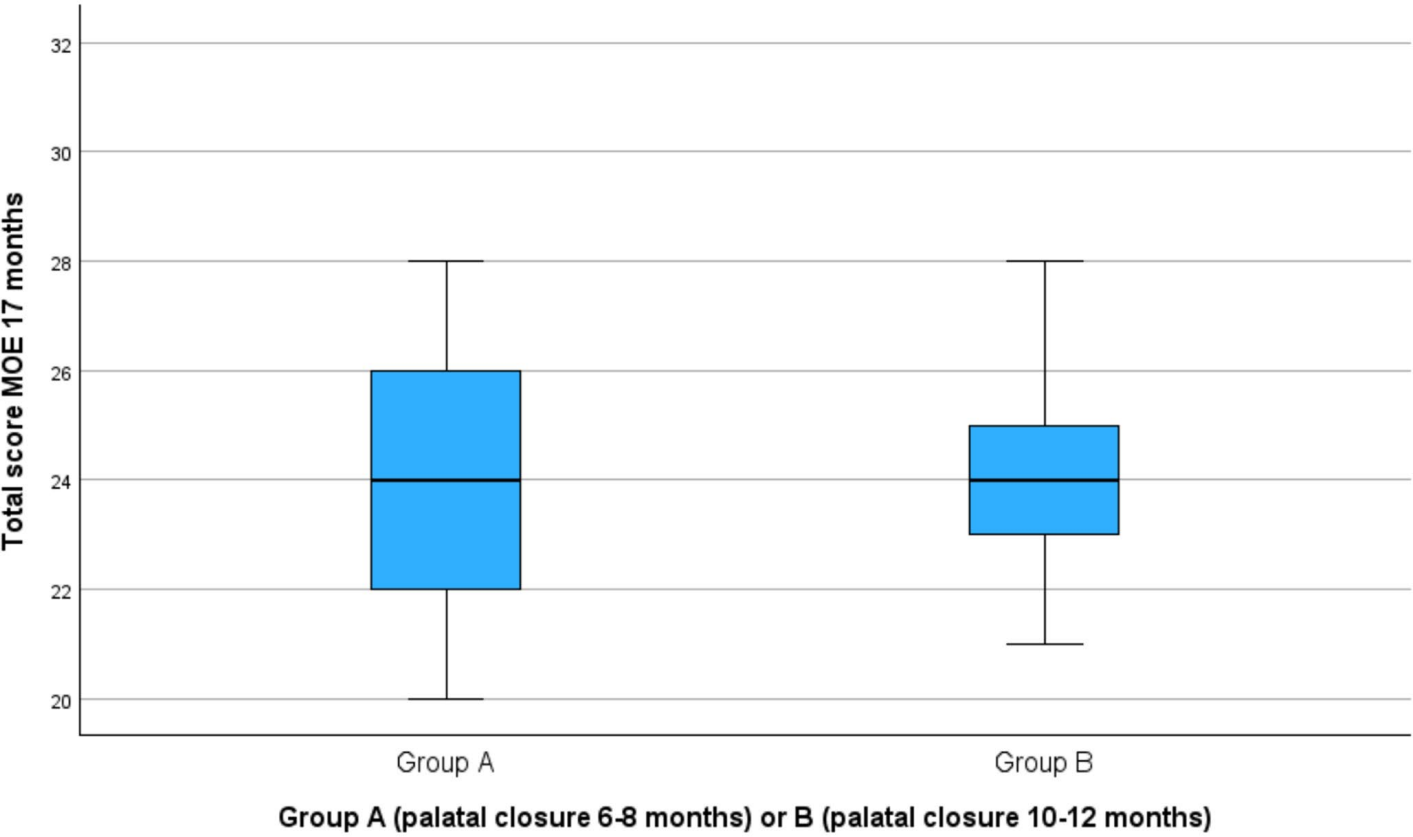
Distribution total MOE score of group A (*n* = 29) and B (*n* = 29) at the age of 17 months

At 17 months, 29 valid recordings were available for each group, with one recording missing in each. Total MOE scores ranged from 20 to 28 in Group A and from 21 to 31 in Group B (Fig. 3). The mean total MOE score was 23.86 (SD = 2.20) for Group A and 24.17 (SD = 2.56) for Group B. No significant difference was observed (t(55) = −0.495, *p* =.623).

However, a significant group difference was found for the *lateral tongue movement* item, favoring Group B (t(56) = −2.03, *p* =.047). The mean difference was − 0.28 (95% CI [−0.55, −0.004]), suggesting better lateral tongue movement in children who underwent palatal closure at 10–12 months.

### Mastication progression over time

**Fig. 4:**
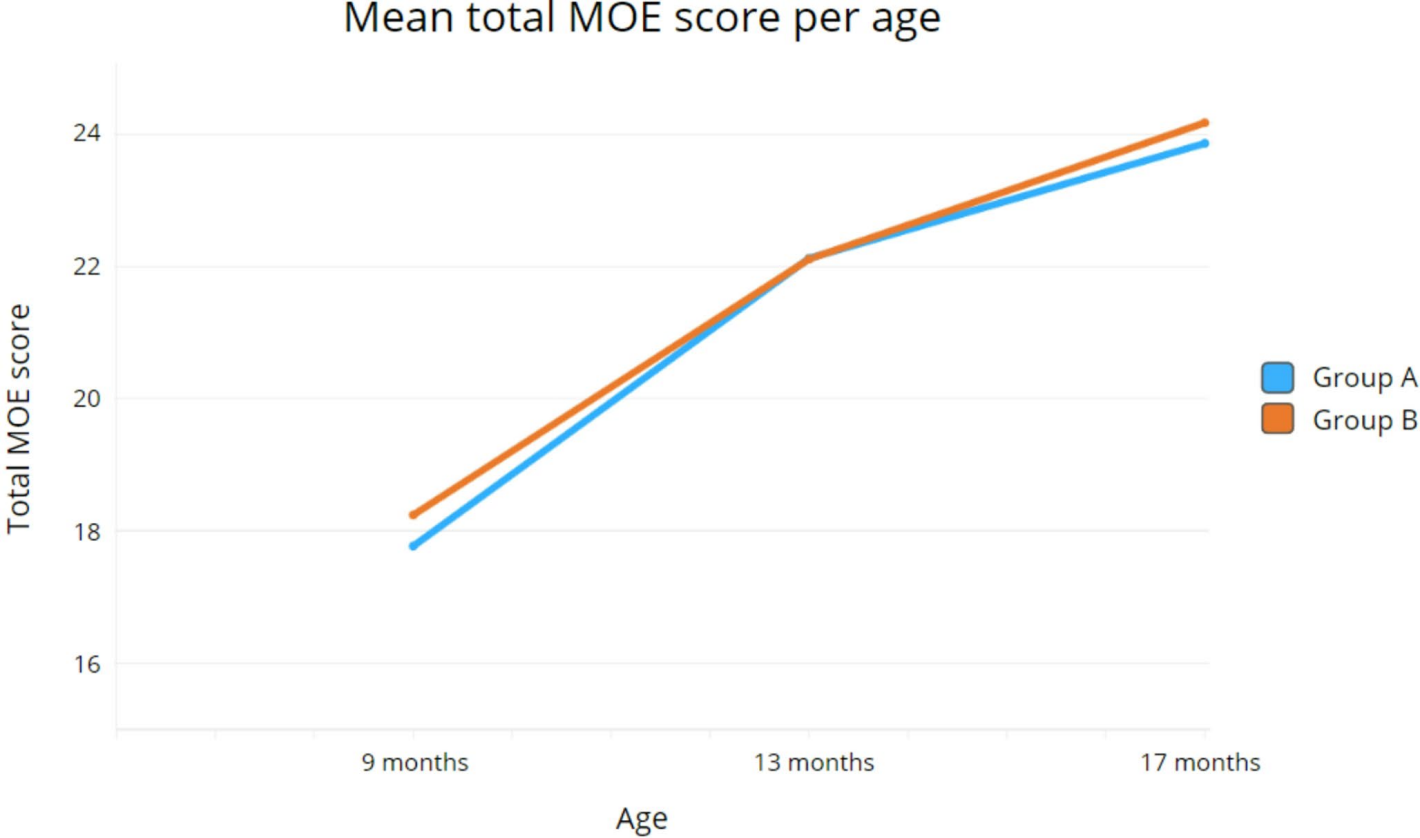
Progression of the mean total MOE score per subject age

Both groups showed clear progression in mastication over time. In Group A, mean total MOE scores increased from 17.77 (SD = 3.19) at 9 months to 22.12 (SD = 2.88) at 13 months and 23.86 (SD = 2.20) at 17 months. In Group B, mean scores rose from 18.24 (SD = 3.52) at 9 months to 22.11 (SD = 3.68) at 13 months and 24.17 (SD = 2.56) at 17 months (Fig. 4).

**Fig. 5:**
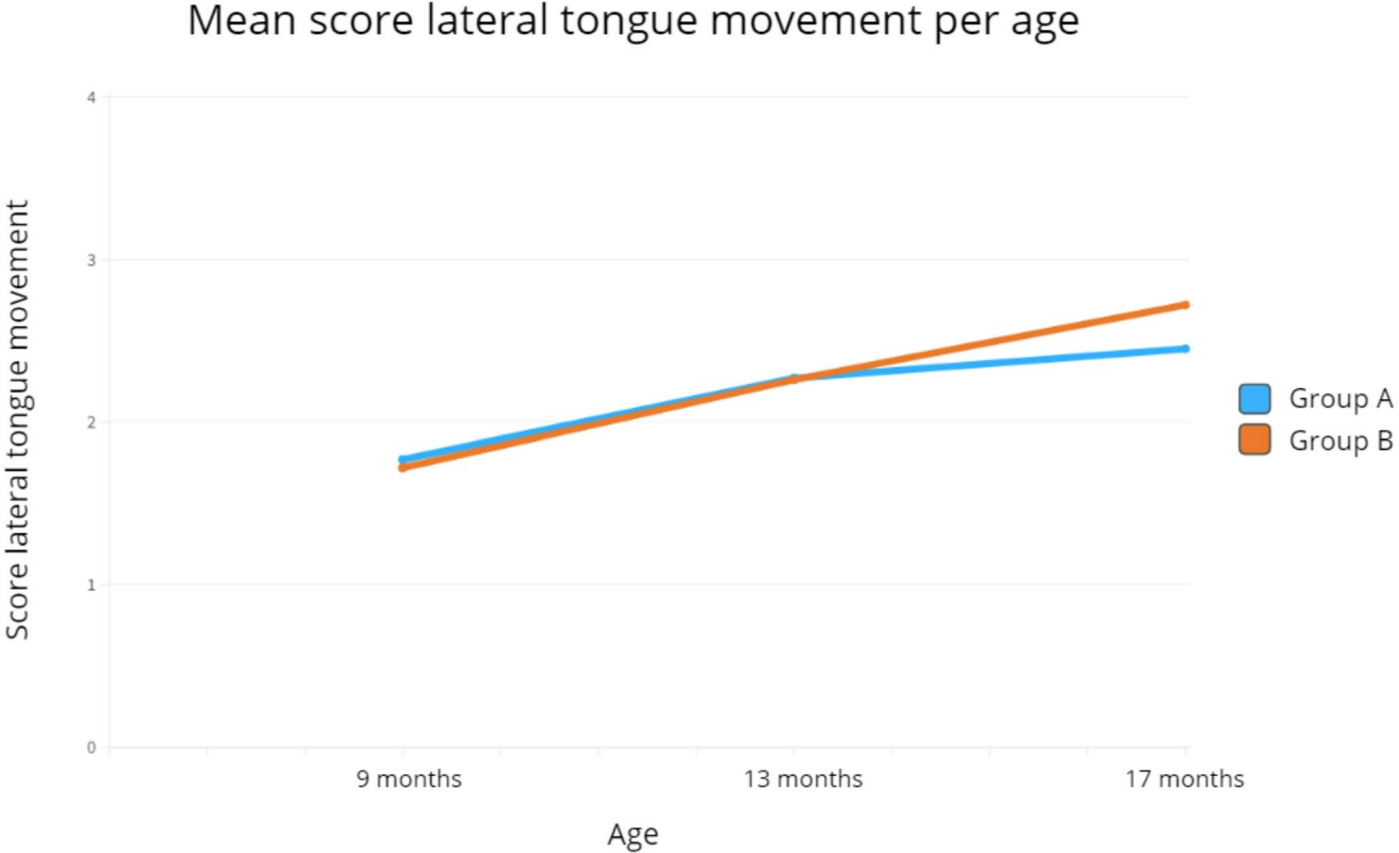
Progression of the mean score of lateral tongue movement per subject age

Improvements in *lateral tongue movement* are shown in Fig. 5. Group A had mean scores of 1.77 (SD = 0.61), 2.27 (SD = 0.72), and 2.45 (SD = 0.51) at 9, 13, and 17 months, respectively. In Group B, scores were 1.72 (SD = 0.46), 2.26 (SD = 0.59), and 2.72 (SD = 0.53), respectively.

### Comparison to healthy children

MOE scores in both cleft groups were compared to those of healthy children reported by Remijn et al. [6]. At 9 months, a significant difference was found (t(14) = − 2.16, *p* =.048), with a mean difference of − 0.24 (95% CI [–0.47, − 0.002]), indicating lower mastication performance in the cleft group. At 13 months (t(14) = − 1.04, *p* =.316) and 17 months (t(14) = − 0.75, *p* =.468), differences were not statistically significant. The mean age of the healthy comparison group was 21.7 months (SD = 12.8) [6].

## Discussion

Mastication is a complex function involving the coordinated activity of orofacial structures under central nervous system control. Alongside breathing, swallowing, sucking, and speech, mastication is one of the key vital functions [26, 27]. Previous studies on mastication in children with cleft palate have mainly examined isolated aspects of chewing, such as jaw movement or tongue function. This study evaluates overall masticatory performance as a functional outcome of surgical treatment. This is relevant, as effective chewing is essential for proper food processing and contributes directly to healthy growth and development [7].

Our findings showed a clear improvement in mastication with age, as reflected in the increasing mean MOE scores between 9 and 17 months. This progression is consistent with the natural course of masticatory development, which begins around 6 months of age and typically advances rapidly by 9 months, when foundational oral motor patterns are established [6, 28]. Between 9 and 13 months, a particularly sharp increase in mastication performance was observed, followed by a more gradual improvement up to 17 months. These findings align with the developmental trajectories described by Remijn et al. [6].

Although we compared MOE scores in children with CL/P to those of a healthy control group, the age difference between samples limits the strength of these comparisons. The results should therefore be interpreted as exploratory. By 17 months, the mastication scores of children with CL/P were no longer significantly different from those of healthy children, although the mean age of the comparison group in Remijn et al. was higher [6]. 

Contrary to our initial hypothesis, we found no statistically significant difference in total MOE scores between the early (6–8 months) and late (10–12 months) palatal closure groups at any of the time points. We had anticipated that earlier closure would facilitate earlier masticatory development. One possible explanation for this unexpected finding is the impact of postoperative recovery on feeding behavior, especially shortly after surgery. Additionally, the difference in clinical status at M1 (i.e., one group post-operative, the other pre-operative) may limit the interpretability of comparisons at that time point.

Interestingly, a significant difference was found in the *lateral tongue movement* item at 17 months, favoring the late closure group (Group B). One possible explanation is that children with an unrepaired palate may develop compensatory tongue movements to direct food away from the cleft during feeding [7]. This compensatory behavior could enhance lateral tongue activity, which was then reflected in higher scores once the palate was repaired.

It is important to note that mastication continues to develop up to approximately four years of age and is influenced by various factors, including tooth eruption and orofacial muscle coordination [6, 7, 29]. Although we observed improvements in mastication over time, we did not systematically record the number or condition of erupted teeth, which may have influenced MOE scores. Future studies should include dental status to better understand its role in mastication. Another noteworthy finding was the significantly higher rate of fistula surgery in Group B compared to Group A. Although this was not a primary outcome of our study, it supports existing literature suggesting that earlier closure may reduce the risk of postoperative complications.

This study has several limitations. First, due to the lack of a validated tool specifically designed for children with clefts, we used the MOE instrument, which was originally developed for other pediatric populations. Although it has been validated for use in young children, its sensitivity to subtle differences in children with clefts remains uncertain. Second, the relatively small sample size and the exclusion of 20 participants due to missing or invalid video data may have reduced the statistical power. Based on our sample size calculation, 80 children (40 per group) were required to detect a clinically relevant difference with 80% power at α = 0.05. However, due to logistical and funding constraints, only 60 children were included in the mastication analysis. Additionally, mastication was only assessed at 9, 13, and 17 months. Given that masticatory development continues beyond this age, these measurements may not fully capture the long-term effects of surgical timing. The decision to stop follow-up at 17 months was based on the original study design and available resources. Nevertheless, to fully understand the developmental trajectory of mastication in this population, future research should extend follow-up to at least four years of age.

Overall, this study provides new insights into the development of mastication in children with cleft palate and the potential influence of surgical timing. While no differences in overall mastication performance were observed between early and late closure groups, differences in specific oral motor functions and postoperative outcomes warrant further investigation. Chewing remains a fundamental but under-researched function in cleft care, and this study provides a foundation for future longitudinal research.

## Conclusion

This study contributes to the limited body of research evaluating overall masticatory function as a treatment outcome in children with cleft palate. Based on assessments at 9, 13, and 17 months using the MOE instrument, no significant differences in total mastication performance were found between early (6–8 months) and late (10–12 months) palatal closure. These findings suggest that earlier closure does not offer a clear advantage in short-term masticatory development. However, timing may influence specific oral motor components, such as lateral tongue movement.

To better understand how children with CL/P compare to their typically developing peers, future studies should include age-matched healthy controls and longer follow-up into later childhood.

## Data Availability

No datasets were generated or analysed during the current study.
